# Predictive models for sepsis in children with *Staphylococcus aureus* bloodstream infections: a retrospective cohort study

**DOI:** 10.1186/s12887-023-04317-2

**Published:** 2023-10-02

**Authors:** Chen Sun, Dongdong Tan, Jiajia Yu, Jingxian Liu, Dihua Shen, Shuang Li, Shiyong Zhao, Liya Zhang, Huajun Li, Kang Cai, Shanshan Xu, Lisu Huang

**Affiliations:** 1 Department of Infectious Disease, Children’s Hospital, Zhejiang University School of Medicine, National Clinical Research Center for Child Health, Hangzhou, Zhejiang 310052 China; 2 Department of Infectious Disease, Xinhua Children’s Hospital, Xinhua Hospital, Shanghai Jiao Tong University School of Medicine, 1665 Kongjiang Road, Shanghai, 200092 China; 3 Department of Pediatrics, Liuzhou Maternity and Child Healthcare Hospital, Liuzhou, Guangxi China; 4 Department of Clinical Laboratory, Xinhua Children’s Hospital, Xinhua Hospital, Shanghai Jiao Tong University School of Medicine, Shanghai, China; 5 Department of Pediatric Surgery, Xinhua Children’s Hospital, Xinhua Hospital, Shanghai Jiao Tong University School of Medicine, Shanghai, China; 6 Department of Infectious Diseases, Hangzhou Children’s Hospital, Hangzhou, China

**Keywords:** *Staphylococcus aureus* bloodstream infections, sepsis, Children, Predictive model

## Abstract

**Background:**

The presence of *Staphylococcus aureus* in the bloodstream can lead to the development of sepsis; however, the severity and risk factors of the systemic inflammatory response to *Staphylococcus aureus* bloodstream infections were unclear. This study is aimed to build a model to predict the risk of sepsis in children with *Staphylococcus aureus* bloodstream infections.

**Methods:**

A retrospective analysis of hospitalized pediatric patients diagnosed with *Staphylococcus aureus* bloodstream infections was performed between January 2013 and December 2019. Each patient was assessed using the pediatric version of the Sequential Organ Failure Assessment score (pSOFA) within 24 h of blood culture collection. A nomogram based on logistic regression models was constructed to predict the risk factors for sepsis in children with *Staphylococcus aureus* bloodstream infections. It was validated using the area under the receiver-operating characteristic curve (AUC).

**Results:**

Of the 94 patients included in the study, 35 cases (37.2%) developed sepsis. The pSOFA scores ranged from 0 to 8, with 35 patients having a pSOFA score of ≥ 2. Six children (6.4%) died within 30 days, who were all from the sepsis group and had different pSOFA scores. The most common organs involved in sepsis in children with staphylococcal bloodstream infections were the neurologic system (68.6%), respiratory system (48.6%), and coagulation system (45.7%). Hospital-acquired infections (adjusted odds ratio [aOR], 3.0; 95% confidence interval [CI], 1.3–7.2), implanted catheters (aOR, 10.4; 95% CI, 3.8–28.4), procalcitonin level ≥ 1.7 ng/mL (aOR, 15.4; 95% CI, 2.7–87.1), and underlying diseases, especially gastrointestinal malformations (aOR, 14.0; 95% CI, 2.9–66.7) were associated with *Staphylococcus aureus* sepsis. However, methicillin-resistant *Staphylococcus aureus* infection was not a risk factor for sepsis. The nomogram had high predictive accuracy for the estimation of sepsis risk, with an AUC of 0.85.

**Conclusions:**

We developed a predictive model for sepsis in children with *Staphylococcus aureus* infection.

**Supplementary Information:**

The online version contains supplementary material available at 10.1186/s12887-023-04317-2.

## Background

Bloodstream infections can lead to prolonged hospital stays and high mortality. *Staphylococcus aureus* is one of the main pathogenic bacteria causing bloodstream infections [1–3]. The incidence of methicillin-resistant *Staphylococcus aureus* (MRSA) infections in children in China is increasing (from 18.0% in 2005 to 30.4% in 2020 ), as in other countries worldwide, and is becoming a major public health problem [3–5].

Previous studies have shown that several risk factors contribute to *Staphylococcus aureus* bloodstream infection-associated deaths in children, including prematurity, low birthweight, congenital heart disease, infective endocarditis, pneumonia, and sepsis [6, 7]. Sepsis is defined as life threatening organ dysfunction due to a dysregulated host response to infection,Which is a strong independent risk factor for mortality from *Staphylococcus aureus* bloodstream infections. Early recognition and treatment of sepsis may substantially improve the prognosis [8–11]. However, the risk factors for sepsis as a complication of *Staphylococcus aureus* bloodstream infections remain unclear.

In this study, we aimed to investigate the clinical features of pediatric sepsis associated with *Staphylococcus aureus* bloodstream infections, and to identify risk factors for sepsis in children with *Staphylococcus aureus* bloodstream infections, to provide a basis for sepsis treatment.

## Methods

### Study cohort and clinical data

A retrospective analysis was conducted on data from a cohort of all pediatric patients (aged < 18 years) diagnosed with *Staphylococcus aureus* bloodstream infections at Xinhua Hospital, a tertiary teaching hospital affiliated with the Shanghai Jiao Tong University School of Medicine in China, between January 2013 and December 2019. It was reviewed and approved by the Ethics Committee of Xinhua Hospital (XHEC-C-2022-014-1). Children with polymicrobial bloodstream infections or incomplete clinical data were excluded. The final cohort comprised 94 patients. Each patient was assessed using the pediatric version of the Sequential Organ Failure Assessment (pSOFA) score. Antimicrobial therapy was considered appropriate if the bacteria identified in the blood culture was susceptible to at least one of the antibiotics administered within 24 h after the collection of culture. Treatment was considered inadequate if the isolated microorganisms were not sensitive to the antibiotics used, as determined by in vitro testing [12].

### Clinical data collection

All medical records of the patients were reviewed by a team of physicians from the microbiology and infectious disease departments. The following variables were collected from the medical charts: demographic characteristics (age, sex, and admission ward), birth status (preterm birth), underlying diseases (congenital gastrointestinal anomalies, congenital heart diseases, and malignancies), probable source of infection, prior hospitalization in the last 6 months, and levels of biomarkers (leukocyte and platelet [PLT] counts, and procalcitonin [PCT], C-reactive protein [CRP], albumin, total bilirubin, creatinine, and lactate levels). These biomarker levels were determined within 24 h of blood culture collection [13]. The severity of organ failure was measured using the age-adapted Sequential Organ Failure score for children (pSOFA). We determined whether sepsis was present in children with *Staphylococcus aureus* bloodstream infections according to the Sepsis-3 criteria [9, 11]. Patients with sepsis were defined as who had a *Staphylococcus aureus* bloodstream infections in the pSOFA score of 2 points in 24 h after the infection. Hospital- or community-onset *Staphylococcus aureus* bloodstream infections was defined as a positive blood culture collected > 48 or ≤ 48 h after presentation, respectively.

### Definitions and microbiological methods

A case of bloodstream infection was defined as positive blood cultures from two separate venipuncture sites. A solitary positive blood culture was considered contaminated and excluded from analysis [14]. Methicillin resistance was defined as an isolate resistant to oxacillin (minimum inhibitory concentration ≥ 4 µg/mL) or cefoxitin (≥ 8 µg/mL). Clinical samples were processed at the microbiology laboratory in accordance with standard operating procedures. Prophylactic antibiotics refer to antibiotics used prior to the onset of *Staphylococcus aureus* bloodstream infection in this retrospective study. If antibiotics are used against *Staphylococcus aureus*, they are considered to have been used appropriately.

### Statistical analysis

**Fig. 1:**
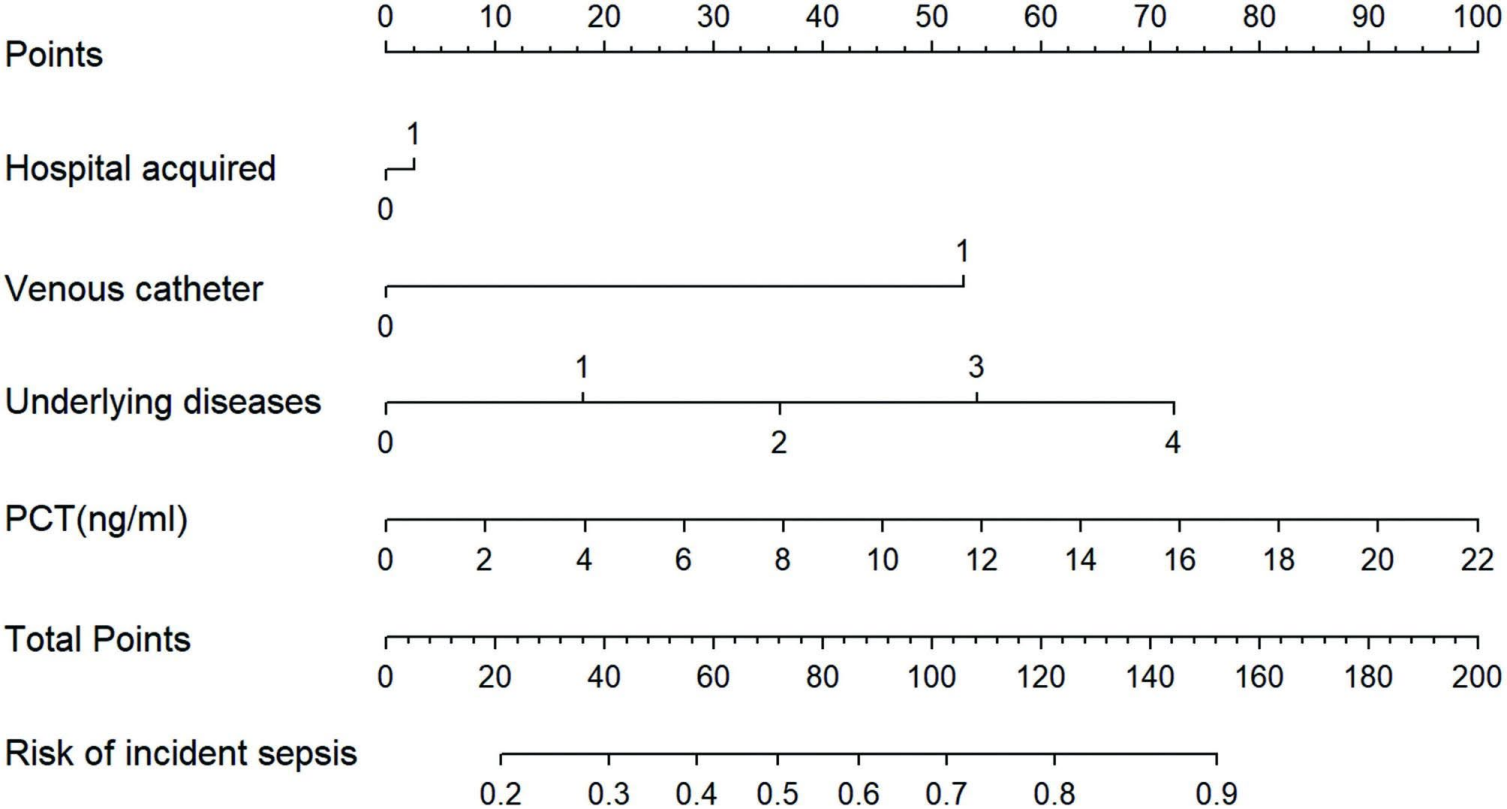
Nomogram for predicting the risk of sepsis in children with *Staphylococcus aureus* bloodstream infections **(a)**. The children’s score for each risk predictor is plotted on the appropriate scale. A total score was calculated by adding each single score. The total points score is plotted on the bottom total points scale. The corresponding value shows the predicted probability of incident sepsis in children with *Staphylococcus aureus* bloodstream infections. The histogram showed the sepsis number in each group. Underlying diseases: none = 0, congenital heart disease = 1, gastrointestinal malformations = 2, tumor = 3, funnel chest or congenital diaphragmatic hernia = 4. HR, hazard ratio; PCT, procalcitonin

Continuous variables with normal distribution are presented as the mean ± standard deviation. Non-normal variables are presented as the median and interquartile range (IQR), and categorical variables are described as frequencies. Chi-square test or Fisher’s exact test were used to compare the categorical variables, and Student’s t-test or the Mann–Whitney U test were used to compare continuous variables. CRP and PCT levels were grouped within the IQR for regression analysis. Logistic regression was used to determine odds ratios (ORs) with 95% confidence intervals (CIs) of complications associated with risk factors of interest among all patients with *Staphylococcus aureus* bloodstream infections for the primary analysis. Sepsis was measured at the onset of *Staphylococcus aureus* bloodstream infections. In the Kaplan-Meier calculation, we assessed the risk of sepsis in children with underlying disease and sepsis is defined as an end event. A nomogram was constructed by proportionally converting each regression coefficient in multivariable logistic regression to a 0 to 100-point scale [15]. The effect of the variable with the highest β coefficient (absolute value) was assigned 100 points. After adding the points across independent variables, the total points were then converted to predicted probabilities. The predictive performance of the nomogram was measured by concordance index and calibrated with 5000 bootstrap samples to decrease the overfit bias. Receiver-operating characteristic (ROC) curves were used to estimate the cutoff value and evaluate the discriminatory ability of the model. Two-tailed P-values < 0.05 were considered statistically significant. R software version 3.6.1 (http://www.r-project.org/) and Empower Stats (www.empowerstats.com; X&Y Solutions, Inc., Boston, MA, USA) were used for all the statistical analyses. The regression coefficient (β) from the multivariable logistic regression model was used to construct the predictive model for sepsis risk as follows:

Logit (Sepsis) = − 2.21 + 0.11×PCT + 1.49×(venous catheter = 1) + 0.06×(hospital-acquired = 1) + 1.46×(underlying diseases = 1) – 1.47×(underlying diseases = 2) – 2.75×(underlying diseases = 3) – 4.51×(underlying diseases = 4) (Fig. 1).

## Results

### Participant characteristics

The study cohort included 94 children with *Staphylococcus aureus* bloodstream infections with a median age of 12 months (IQR: 0.9–51.0 months). Prophylactic antibiotics were used in all patients, of which 59 (63.0%) were used appropriately. Sepsis occurred in 35 children (37.2%); 6 children (6.4%) died within 30 days of diagnosis with *Staphylococcus aureus* bloodstream infections, all from the sepsis group. The mortality of the sepsis group was 17.1%. Only 43 (45.7%) cases had their onset in the hospital. A total of 42 (44.7%) MRSA strains were isolated, including 20 community-acquired MRSA and 22 hospital-acquired MRSA, and 52 (55.3%) were methicillin-sensitive *Staphylococcus aureus* (MSSA) strains, including 31 were community-acquired MSSA strains and 21 hospital-acquired MSSA strains.

### Clinical characteristics

**Fig. 2:**
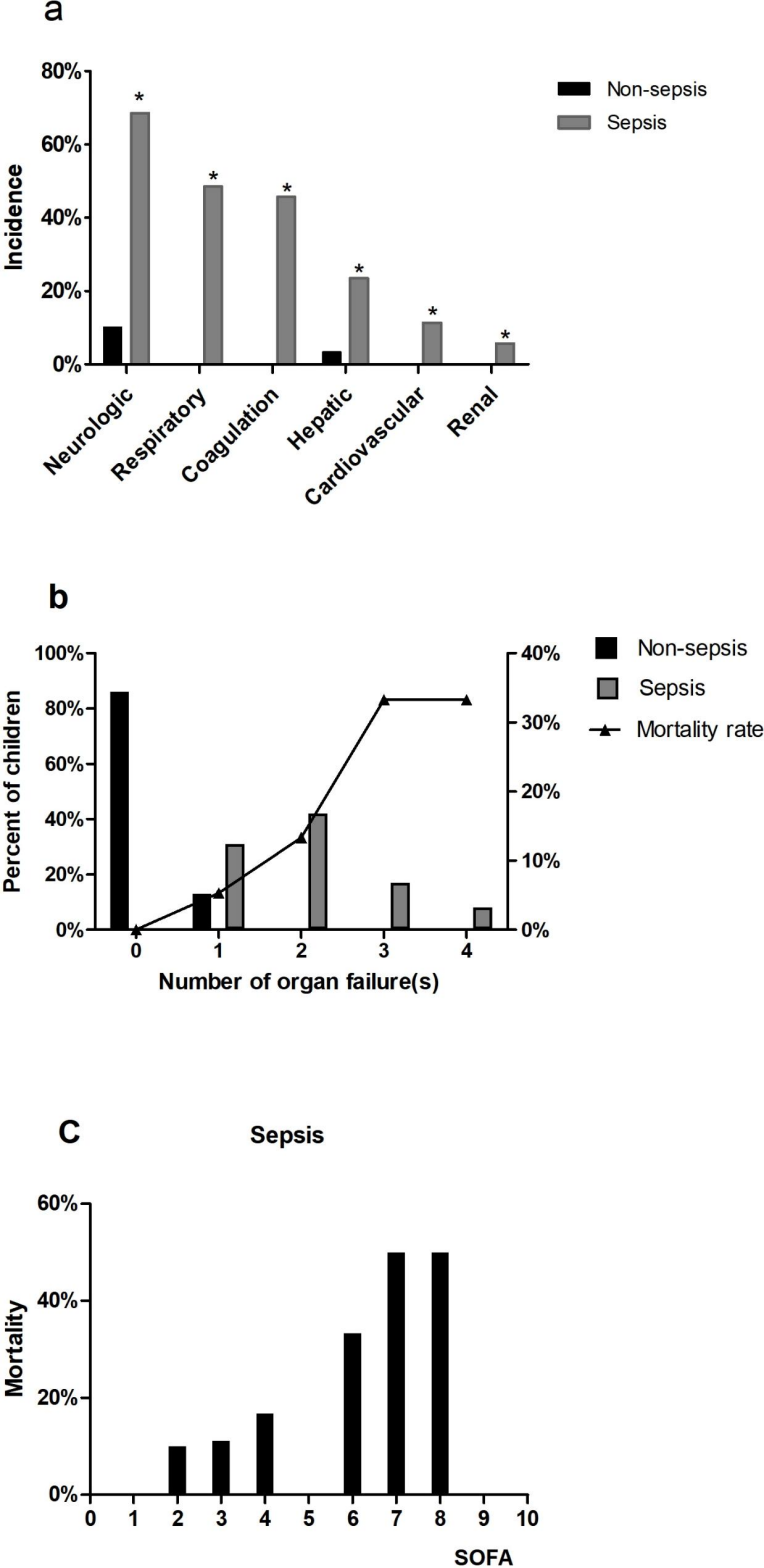
Evaluation of organ dysfunction in children with *Staphylococcus aureus* bloodstream infections. **(a)** Involvement of different systems stratified by sepsis status; **(b)** number of organ failure**(s)** by sepsis status; **(c)** mortality rate in the sepsis and non-sepsis groups according to the pSOFA score. pSOFA, pediatric Sequential Organ Failure Assessment

Differences in hospital-acquired infections and venous catheters were observed between the sepsis and non-sepsis groups. Children with underlying diseases, such as congenital heart disease (26.0%), gastrointestinal malformations (23.0%), and cancer (29.0%), were more likely to develop sepsis than those without. Children in the sepsis group were primarily from the pediatric intensive care unit (34.0%). Children with sepsis had significantly longer hospital stays with a median length of 19 days (IQR, 0.9–37.5 days) compared with those without sepsis (Table 1). In terms of laboratory-test indicators, children in the sepsis group had lower hemoglobin level and platelet count, and higher PCT than those in the non-sepsis group (Table 2). The most common organs involved in sepsis in children with *Staphylococcus aureus* bloodstream infections were the neurologic system (68.6%), respiratory system (48.6%) and coagulation system (45.7%); and two or more organs and systems were involved in 24 patients. Mortality in sepsis was higher in patients with multiorgan failure than in those with single-organ failure. (Fig. 2).

**Table 1:** Participant characteristics according to sepsis development in children with *Staphylococcus aureus* bloodstream infections

Variables	Total	Sepsis	Non-sepsis	*P* value
	(n = 94)	(n = 35)	(n = 59)	
Boys, n (%)	59 (62.8)	20 (57.1)	39 (66.1)	0.39
Age (months), median (IQR)	12.0 (0.9–51.0)	12.0 (2.0–41.5)	12.0 (0.6–58.0)	0.38
Preterm birth, n (%)	8 (8.5)	4 (11.4)	4 (6.8)	0.44
Prior hospitalization within 6 months, n (%)	52 (55.3)	16 (45.7)	36 (61.0)	0.15
Surgical history within 2 weeks, n (%)	25 (26.6)	11 (31.4)	14 (23.7)	0.41
Hospital-acquired infection, n (%)	43 (45.7)	21 (35.6)	22 (62.9)	0.01
Venous catheter, n (%)	35 (37.2)	24 (68.6)	11 (18.6)	< 0.01
MRSA, n (%)	42 (44.7)	17 (48.6)	25 (42.4)	0.56
Underlying diseases n (%)	46 (48.9)	28 (60.9)	18 (39.1)	< 0.01
Congenital heart disease, n (%)	16 (17.0)	9 (25.7)	7 (11.9)	
Gastrointestinal malformation, n (%)	12 (12.8)	8 (22.9)	4 (6.8)	
Tumor, n (%)	16 (17.0)	10 (28.6)	6 (10.2)	
Other, n (%)	2 (2.1)	1 (2.9)	1 (1.7)	
Source of bloodstream infection				0.07
Skin and soft tissue infection, n (%)	39 (41.5)	15 (42.9)	24 (40.7)	
Primary bacteremia, n (%)	15 (16.0)	6 (17.1)	9 (15.3)	
Pneumonia, n (%)	19 (20.2)	11 (31.4)	8 (13.6)	
Osteomyelitis, n (%)	12 (12.8)	1 (2.9)	11 (18.6)	
Other sources, n (%)	9 (9.6)	2 (5.7)	7 (11.9)	
Prophylactic antibiotics, n (%)	59 (62.7)	24 (68.6)	35 (59.3)	0.37
Length of hospital stay, days, median (IQR)	19.0 (9.0–37.5)	29.0 (13.0–47.0)	16.0 (8.5–30.0)	< 0.01

IQR, interquartile range; MRSA, methicillin-resistant *Staphylococcus aureus*

**Table 2:** Laboratory predictors of sepsis in children with *Staphylococcus aureus* bloodstream infections

Laboratory predictor^a^	Total	Sepsis	Non-sepsis	*P* value
	(n = 94)	(n = 35)	(n = 59)	
WBC, median (IQR),10^9^/L	12.2 (7.4–17.9)	9.4 (5.1–16.6)	12.6 (8.9–18.1)	0.37
ANC, median (IQR), 10^9^/L	8.1 (4.0–12.8)	5.8 (3.5–13.3)	8.5 (5.0–11.4)	0.95
HGB, median (IQR), g/L	113.0 (100.0–129.0)	107.0 (88.0–124.0)	118.0 (105.0–133.0)	< 0.01
PLT, median (IQR), 10^9^/L	287.5 (208.2–382.5)	162.0 (86.0–286.5)	307.0 (259.0–429.5)	< 0.01
CRP, median (IQR), mg/L	28.0 (4.0–68.5)	34.0 (5.8–78.5)	20.0 (4.0–65.0)	0.83
PCT, median (IQR), ng/mL	0.6 (0.2–1.7)	1.2 (0.5–6.9)	0.3 (0.1–1.0)	< 0.01
ALT, median (IQR), u/L	25.0 (17.0–32.0)	27.0 (20.0–33.5)	23.5 (16.2–32.0)	0.23
ALB, median (IQR), g/L	35.5 (32.0–38.8)	36.8 (32.2–38.6)	35.0 (31.4–39.5)	0.76
TBIL, median (IQR), µmol/L	12.1 (6.5–31.5)	16.0 (8.7–27.1)	8.8 (6.1–85.7)	0.08
Cr, median (IQR), µmol/L	28.0 (21.4–35.8)	26.0 (20.9–35.4)	28.9 (22.2–35.9)	0.79

^a^ All laboratory tests were performed within 24 h after the collection of blood culture samples

ALB, albumin; ALT, alanine aminotransferase; ANC, absolute neutrophil count; Cr, creatinine; CRP, C-reactive protein; HGB, hemoglobin; IQR, interquartile range; PCT, procalcitonin; PLT, platelet count; TBIL, total bilirubin; WBC, white blood cell

### Risk factors for sepsis

**Fig. 3:**
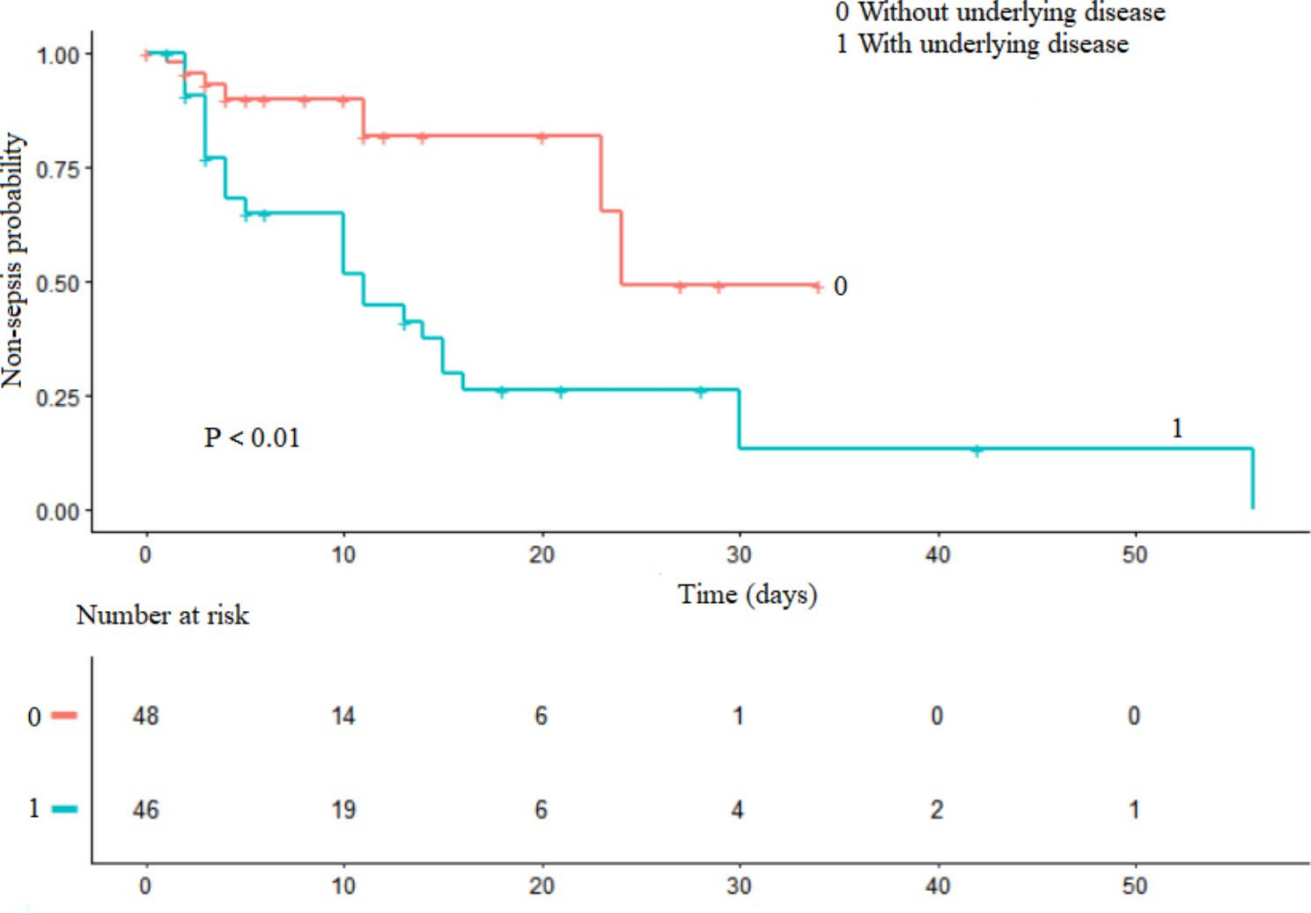
Kaplan-Meier survival analysis of sepsis probability. Sepsis probability according to underlying disease. The abscissa is the time from admission to sepsis, and the ordinate is the probability of sepsis. 0: Without underlying disease; 1: With underlying disease

Logistic regression analysis revealed that catheter implantation, hospital-acquired infections, underlying disease, and elevated PCT level were associated with sepsis development. Prophylactic antibiotics, MRSA and source of infection did not contribute to sepsis risk (Table 3). Sepsis risk was three times higher in patients with hospital-acquired infections than in those with community-acquired infections (adjusted odds ratio [aOR], 3.0; 95% CI, 1.3–7.2). Patients with catheter implantation had a 10.4 times higher risk of sepsis than those without catheter implantation (aOR, 10.4; 95% CI, 3.8–28.4). Compared with those without underlying disease, sepsis risk was 6 times higher in children with congenital heart disease (aOR, 7.0; 95% CI, 1.8–27.1), 13 times higher in those with gastrointestinal malformations (aOR, 14.0; 95% CI, 2.9–66.7), and 10 times higher in those with tumors (aOR, 11.1; 95% CI, 2.9–42.0). Additionally, PCT ≥ 1.70 ng/mL (aOR, 15.4; 95% CI, 2.7–87.1) was a risk factor for sepsis in children with *Staphylococcus aureus* bloodstream infections. The Kaplan-Meier curve displayed the sepsis probability in children with *Staphylococcus aureus* bloodstream infections was related to the underlying diseases (Fig. 3).

**Table 3:** Risk factors associated with sepsis among children with *Staphylococcus aureus* bloodstream infections

Variable	n (%)	OR (95% CI)	*P* value	Adjusted OR (95% CI)^a^	*P* value
Preterm birth	8 (8.5)	1.8 (0.4–7.6)	0.44	1.5 (0.3–6.8)	0.58
Hospitalization within the past 6 months	52 (55.3)	0.5 (0.2–1.3)	0.15	0.5 (0.2–1.2)	0.12
Surgery within the past 2 weeks	25 (26.6)	1.5 (0.6–3.7)	0.42	1.4 (0.6–3.7)	0.45
Venous catheter	35 (37.2)	9.5 (3.6–25.1)	< 0.01	10.4 (3.8–28.4)	< 0.01
Hospital-acquired infection	43 (45.7)	3.1 (1.3–7.3)	0.01	3.0 (1.3–7.2)	0.01
MRSA	42 (44.7)	1.3 (0.6–3.0)	0.56	1.2 (0.5–2.8)	0.70
Prophylactic antibiotics	59 (62.8)	1.5 (0.6–3.6)	0.37	1.8 (0.7–4.5)	0.22
Underlying diseases					
None	48 (51.1)	1.0 (reference)		1.0 (reference)	
Congenital heart disease	16 (17.0)	7.5 (2.1–26.9)	< 0.01	7.0 (1.8–27.1)	< 0.01
Gastrointestinal malformation	12 (12.8)	11.7 (2.8–49.6)	< 0.01	14.0 (2.9–66.7)	< 0.01
Cancer	16 (17.0)	9.8 (2.7–35.5)	< 0.01	11.1 (2.9–42.0)	< 0.01
Other	2 (2.1)	5.9 (0.3–104.9)	0.23	7.9 (0.4–151.0)	0.17
Source of bloodstream infections					
Skin and soft tissue infection	39 (41.5)	1.0 (reference)		1.0 (reference)	
Primary bacteremia	15 (16.0)	1.1 (0.3–3.6)	0.92	1.0 (0.3–3.6)	0.94
Pneumonia	19 (20.2)	2.2 (0.7–6.7)	0.17	2.5 (0.7–8.4)	0.26
Osteomyelitis	12 (12.8)	0.1 (0.0–1.2)	0.08	0.1 (0.0–1.1)	0.06
Others	9 (9.6)	0.5 (0.1–2.5)	0.37	0.5 (0.1–2.7)	0.42
PCT (ng/mL) ^b^					
< 0.2	19 (26.0)	1.0 (reference)		1.0 (reference)	
0.2–1.7	34 (46.6)	6.7 (1.3–33.7)	0.02	6.5 (1.3–32.9)	0.02
≥ 1.7	20 (27.4)	15.8 (2.8–89.0)	< 0.01	15.4 (2.7–87.1)	< 0.01

^a^Adjusted for age and sex; ^b^PCT level was grouped according to the IQR.

CI, confidence interval; IQR, interquartile range; MRSA, methicillin-resistant *Staphylococcus aureus*; OR, odds ratio; PCT, procalcitonin

### Predictive performance of the nomogram

**Fig. 4:**
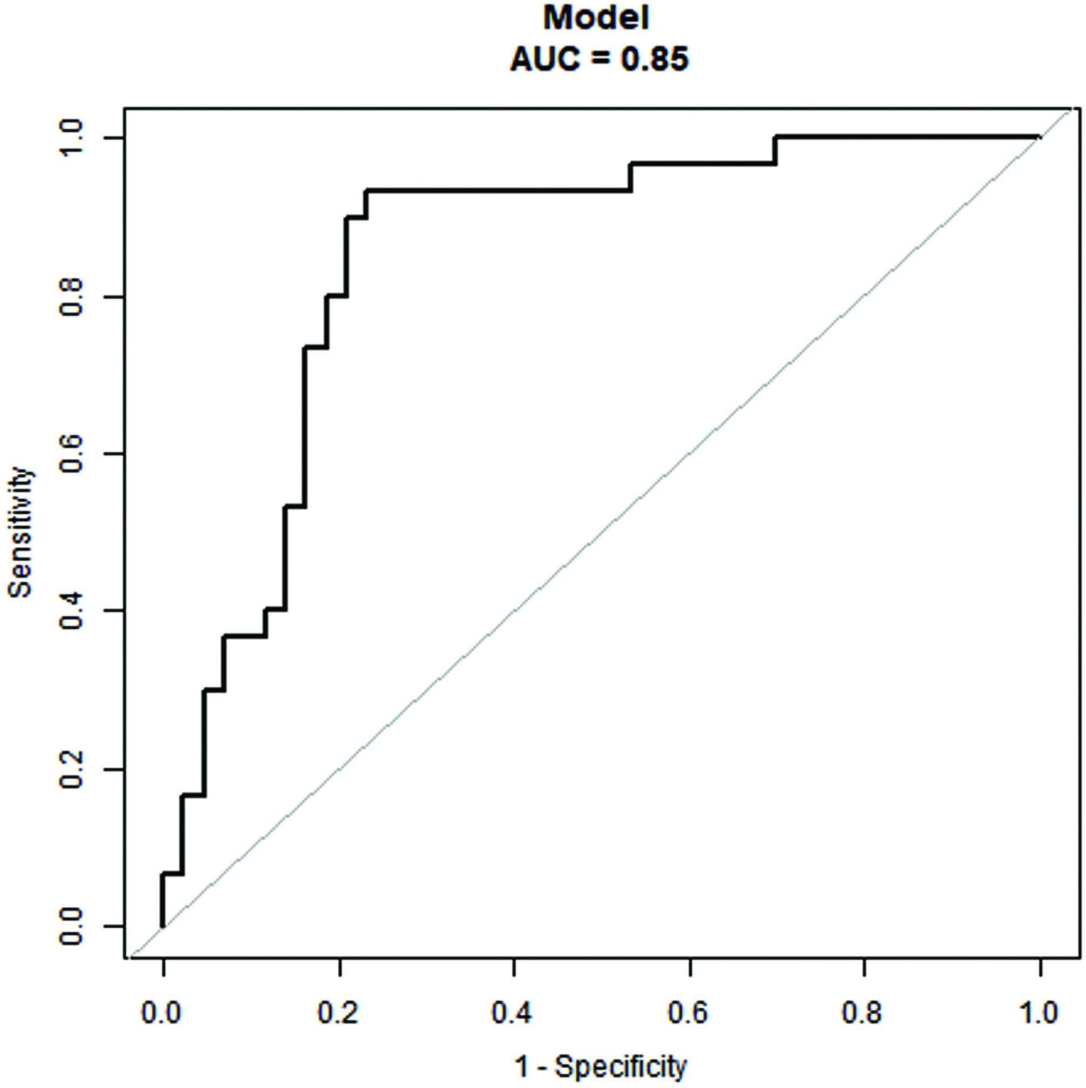
Area under the receiver-operating characteristic curve (AUC). The AUC of the model from observed data (nomogram) was 0.85. The cutoff value was − 0.71 with a sensitivity of 93.3% and a specificity of 76.7%

Using the regression discontinuity analysis, we identified the breakpoints of nomogram points as -0.71. Nomogram performance was measured by the area under ROC curve (AUC), and the AUC of the model was 0.85 (95% CI, 0.76–0.94). The cutoff score was − 0.71, with a sensitivity of 93.3% and a specificity of 76.4% (Fig. 4). Its predictive accuracy was also measured using the bootstrap (5000 resample) method, and the AUC remained largely unchanged (AUC, 0.85) (Fig. 4).

## Discussion

This study explored the clinical characteristics of children with *Staphylococcus aureus* bloodstream infections at a large tertiary care children’s hospital, particularly the risk factors they had for developing sepsis. Sepsis occurred in approximately a third of the pediatric patients with *Staphylococcus aureus* bloodstream infections.The pSOFA scores varied among the six children who died of sepsis. The number of organ failures and SOFA scores were closely related to mortality. Interestingly, unlike some previous studies [2, 6, 16], we found that hospital-acquired infections, underlying diseases, implanted catheters, and elevated PCT levels were associated with sepsis among children with *Staphylococcus aureus* bloodstream infections, and MRSA was not identified as a risk factor.

Bacterial bloodstream infections is one of the most common infectious diseases in children. The outcome of *Staphylococcus aureus* bloodstream infections or staphylococcal sepsis is influenced by the characteristics of the patient (e.g., age, immunologic status, and comorbidities) and those of the infection (e.g., pathogen type, virulence, site of infection, and inoculum) [16]. In our study, the proportion of sepsis complicated by *Staphylococcus aureus* bloodstream infections was relatively high (37.2%). Liu et al. [17] found that Group B *Streptococcus* was the most common bloodstream infection bacterial pathogen among neonates in the neonatal intensive care unit with early-onset sepsis, accounting for 14.6% of bloodstream infections. The mortality of pediatric sepsis ranges from 4.0 to 50.0%, depending on the severity of the illness, risk factors, and geographic location [18]. The mortality of sepsis in children with bloodstream infections differs according to the pathogen type. Li et al. [19] found a 22.6% mortality among young children with *Klebsiella pneumoniae* bloodstream infections. A prospective multicenter study has found that the mortality rates of sepsis or septic shock in adult patients with *Staphylococcus aureus* bloodstream infections was 38.0–86.0%, and that sepsis was strongly associated with poor outcomes [20]. In our study the mortality rate among children with staphylococcal sepsis was much higher than that reported for children with sepsis in a children’s hospital in the United States (6.7%) [21]. In adult patients with suspected infections, the Sepsis-3 Task Force validated the SOFA score, and they discovered that it was either on par with or better than other scoring systems at differentiating in-hospital mortality [11]. The SOFA score has a number of significant drawbacks, including the fact that it was created for adult patients and that the measurements it uses have a wide range of age-appropriateness. The best SOFA thresholds for children are currently the subject of much research [9]. The pSOFA scores of the six children who died of sepsis in this study were different. The outcomes can be more biased in a big sample. The majority of children who died from sepsis had multiple-organ dysfunction syndrome [18]. Each organ injury contributes to the patient’s risk of death, with complex interrelationships between systems [22]. In this study a higher number of organ failures was associated with a higher mortality rate, similar to the findings of a retrospective study [21].

The emergence of MRSA makes clinical antibiotic treatment a great challenge [23]. *Staphylococcus aureus* is one of the pathogens that have a high resistance rate and are most likely to fail prophylactic antibiotic therapy [24]. MRSA can prolong the duration of bloodstream infections and increase the likelihood of sepsis [2, 16]. MRSA can cause various infections that are primarily attributed to the presence of extracellular and surface virulence factors, such as Panton–Valentine leucocidin (PVL) [25], which is associated with an increased risk of sepsis. However, MRSA was not a risk factor for sepsis in in this study. Further interpretation of these results may require molecular typing and strain virulence identification. Moreover, our results suggest that prophylactic antibiotic use has little effect on sepsis in children with *Staphylococcus aureus* bloodstream infections.

Sepsis is more common in children with underlying medical conditions, especially those with congenital heart disease, cancer, and gastrointestinal malformations. Children with underlying diseases often have concomitant immune deficiency and are frequently exposed to invasive surgery that may increase risk of sepsis [26–28]. The mortality rate of sepsis in patients with underlying diseases is significantly higher than in previously healthy children [29]. Children with intestinal malformations are prone to gastrointestinal dysfunction, such as paralytic ileus. Intestinal obstruction induces a shift in gastrointestinal bacteria, thereby increasing the risk of sepsis. Congenital gastrointestinal malformation is a risk factor for death in children with sepsis [30]. The presence of a long-term intravascular catheter is associated with serious hematogenous complications in patients with *Staphylococcus aureus* bloodstream infections [31], consistent with our results. The present study showed that in children with *Staphylococcus aureus* bloodstream infections, hospital-acquired infections were more likely than community-acquired infections to result in sepsis (*p* = 0.01). In our study, children were primarily admitted to the intensive care unit and had intravenous catheters and long hospital stays, which increased the risk of hospital-acquired infections and sepsis [32].

Compared with several other bloodstream biomarkers, PCT is the current standard for the identification of bacterial infections because of its wide biological range, short induction time after bacterial stimulus, and long half-life [33–36]. A meta-analysis has suggested a cutoff of between 1.0 and 2.0 ng/mL PCT to distinguish patients with sepsis from those with other inflammatory conditions [36]. In this study, logistic regression analysis showed that elevated PCT was a risk factor for sepsis in children with *Staphylococcus aureus* bloodstream infections. The risk of sepsis was higher in children with a PCT level ≥ 1.7 ng/mL.

This study has several limitations. First, as a single-center retrospective cohort study, it was not possible to analyze the long-term trends associated with the study variables. Nevertheless, although the patient population may not be representative of all children with *Staphylococcus aureus* bloodstream infections, some assistance was provided to children in hospitals, particularly those with underlying illnesses, while they were being diagnosed and treated. Second, Due to the retrospective design, it was not possible to detect the virulence and resistance characteristics of *Staphylococcus aureus*, such as PVL virulence genes. Ultimately, the tiny sample size prevented us from validating the model. These findings, however, have offered some direction for additional study and should be confirmed by subsequent research using larger sample sizes.

## Conclusions

In conclusion, sepsis is a serious medical complication of *Staphylococcus aureus* bloodstream infections. The development of sepsis is closely related to underlying diseases and other risk factors, especially gastrointestinal malformations. Further investigations are needed for the molecular typing and virulence characterization of *Staphylococcus aureus*. Therefore, clinicians can optimize the treatment plan according to these risk factors and drug sensitivity results, minimize unnecessary invasive procedures, control the emergence of multidrug resistant strains, and reduce the mortality rate and poor prognosis rate of *Staphylococcus aureus* bloodstream infections.

### Electronic supplementary material

Below is the link to the electronic supplementary material.

**Supplementary file 1.** Raw data related to the study

**Supplementary file 2.** The code used for the Nomogram analysis

## Data Availability

The datasets used and/or analyzed during the current study are available from the corresponding author on reasonable request.

## Abbreviations

AOR: Adjusted odds ratio
AUC: Area under the curve
CI: Confidence interval
CRP: C-reactive protein
IQR: Interquartile range
MRSA: Methicillin-resistant *Staphylococcus aureus*
MSSA: Methicillin-sensitive *Staphylococcus aureus*
OR: Odds ratio
PCT: Procalcitonin
PLT: Platelets
pSOFA: Pediatric Sequential Organ Failure Assessment
ROC: Receiver-operating characteristic
