# Supplementary material for: Predictive models for sepsis in children with Staphylococcus aureus bloodstream infections: a retrospective cohort study
Source: BMC Pediatr. 2023 Oct 2;23:496. doi: 10.1186/s12887-023-04317-2 (PMC10544563; doi:10.1186/s12887-023-04317-2)
Supplement: Supplementary file 2 — The code used for the Nomogram analysis [file 12887_2023_4317_MOESM2_ESM.pdf]

Supplementary file 2. The code used for the Nomogram analysis

We did the statistical analysis with R and Empower Stats.

```
```{r setup, include=FALSE}
knitr::opts_chunk$set(echo = TRUE,warning=FALSE,message=FALSE)
library(pacman)
p_load(openxlsx,plyr,pillar,rlang,grid,vcd,vcdExtra,knitr,dplyr,grid,formattable,ggplot2,doBy,
rcompanion,tableone,kableExtra,performance,see,MASS,caTools,ROCR,Hmisc,grid,lattice,
Formula,ggplot2, rms)
```
```{r}
setwd("C:/Users/wuyuhou/Desktop")
raw <- read.xlsx("11.29.xlsx")
str(raw)
names(raw) = c("ID","HospitalID","age","Sepsis","Hospital.acquired",
                    "Venous.catheter","Underlying.diseases" ,"PCT")
```
```{r}
raw$Hospital.acquired = as.factor(raw$Hospital.acquired)
raw$Venous.catheter = as.factor(raw$Venous.catheter)
raw$Underlying.diseases = as.factor(raw$Underlying.diseases)
```
```{r,fig.align='center',fig.width=5,fig.height=3}
model_1=glm(sep ~ cvc+ha+pct+basic,data=raw,family = binomial(link = "logit"))
summary(model_1)
model_2=glm(sep ~ age+cvc+ha+pct+basic,data=raw,family = binomial(link = "logit"))
performance_roc(model_1,model_2)
plot(performance_roc(model_1,model_2))
```
```{r,fig.align='center',fig.width=4}
dd=datadist(raw)
options(datadist="dd")
var.labels = c(ID="ID",
                    Hospital ID="Hospital ID",age="age",Sepsis="Sepsis",
                    Hospital.acquired="Hospital acquired",Venous.catheter = "Venous catheter",
                    Underlying.diseases="Underlying diseases",PCT = "PCT(ng/ml)")
label(raw) = lapply(names(var.labels),
                        function(x) label(raw[,x])=var.labels[x])
```
```

```
f1 <- lrm(Sepsis ~ Hospital.acquired+Venous.catheter+Underlying.diseases+PCT, data = raw)
nom <- nomogram(f1, fun= function(x)1/(1+exp(-x)), # or fun=plogis
                 lp=F, funlabel="Risk of incident sepsis")
plot(nom, force.label=TRUE,vnams="labels")
```
